# Supplementary figures and images for: Computational drug repurposing against SARS-CoV-2 reveals plasma membrane cholesterol depletion as key factor of antiviral drug activity
Source: PLoS Comput Biol. 2022 Apr 11;18(4):e1010021. doi: 10.1371/journal.pcbi.1010021 (PMC9022874; doi:10.1371/journal.pcbi.1010021)

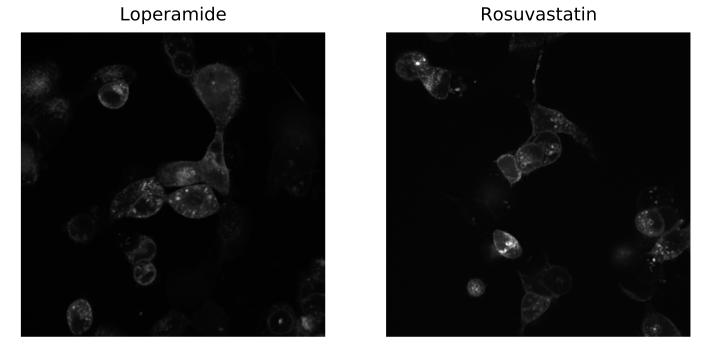

Supplement: S1 Fig — (TIFF) [file pcbi.1010021.s001.tiff]

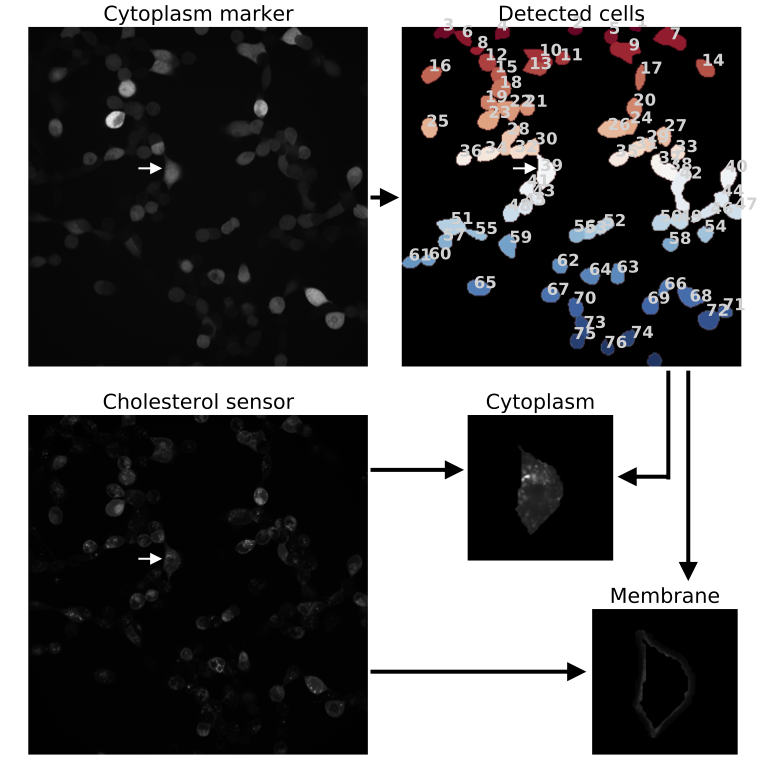

Supplement: S2 Fig — Cells are detected on the cytoplasm marker channel, then boundaries of cytoplasm and membrane are determined for each cell. The D4H channel is used for the calculation of the PM/IC ratio. (TIFF) [file pcbi.1010021.s002.tiff]

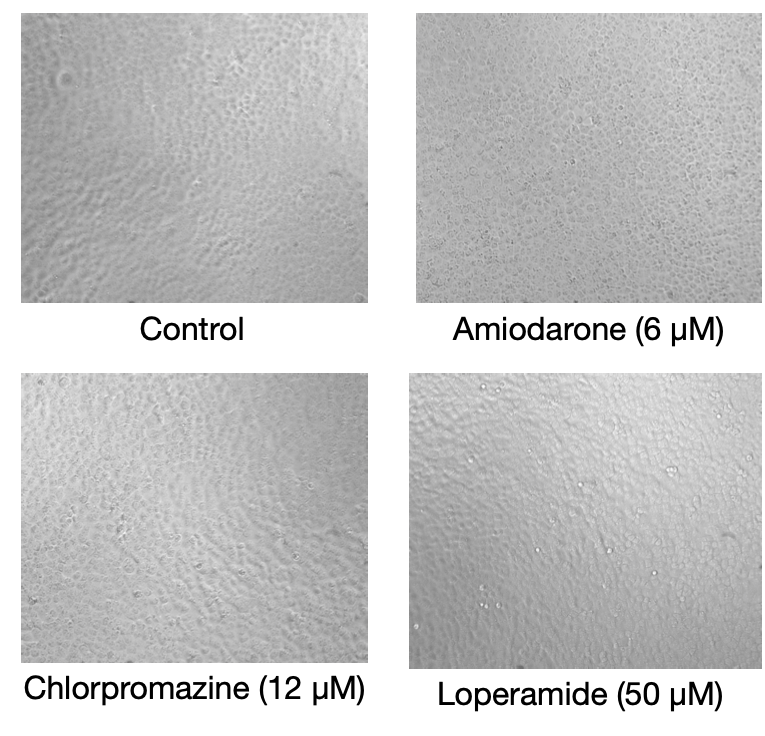

Supplement: S3 Fig — (TIFF) [file pcbi.1010021.s003.tiff]
