# Supplementary material for: Computational drug repurposing against SARS-CoV-2 reveals plasma membrane cholesterol depletion as key factor of antiviral drug activity
Source: PLoS Comput Biol. 2022 Apr 11;18(4):e1010021. doi: 10.1371/journal.pcbi.1010021 (PMC9022874; doi:10.1371/journal.pcbi.1010021)
Supplement: Attachment — Submitted filename: Barsi_SARSCoV2_PLOSCOMPBIO_answers.docx [file pcbi.1010021.s005.docx]

### Reviewer #1

**Comments to the Authors:**

**In this article, the authors introduced an interesting and counterintuitive technique for drug repurposing in for SARS-Cov-2: finding drugs that have signature similar to infection signature. The rationale is that infected cells activate adaptive, antiviral pathways, so that the relationship between effective drug and disease signature can be more ambiguous. In addition, as infected cells activate adaptive antiviral pathways, inhibiting these pathways does not decrease viral replication.**

**Based on the proposed analysis in this paper, three drugs (loperamide, amiodarone, and chlorpromazine) were identified as helpful in decreasing the plasma membrane cholesterol level, which in turn can have antiviral effects.**

**The authors have provided sufficient analysis and experiments to support their claim. In addition, the manuscript is well-written.**

We thank the reviewer for the thoughtful comments, positive feedback, and highlighting the importance of the relationship between effective drug-induced and disease signatures.

**I only have three minor comments that would help to improve the quality of the article:**

**1. The authors used Random Forest Classifier to predict the effective drugs with the 100 trees. It would be great if the authors can provide justification why the choose RF over many other techniques? Also, why did they choose 100 as the number of trees? Would the conclusion be the same if they use other classification techniques or parameters?**

Random Forest Classifier is a widely used classifier, with reasonable performance and established methods to analyse feature importances, thus it is frequently used as a first line ML model.

We used the Python *scikit-learn* library with the default number of estimators (100). With these default parameters, we could reach comparable performance to the unsupervised, similarity-based approach. We agree with the reviewer that a better performance can be achieved if the parameters of the algorithm are optimised. We optimised the number of estimators in 100 different training sets and investigated the performance as their average ROC AUC values. Based on the results of this hyperparameter optimization, we have chosen to run the predictions with 300 trees, and updated the Figure 3C, 3D and the Methods section accordingly.

Importantly, we found that the most important features are not affected by changing the number of estimators of the random forest model, namely SREBF1, SREBF2 and TFAP2A remained the most important features.

The following table shows the top 10 most important features in the case of different numbers of estimators (columns: number of estimators, rows: top 10 features in decreasing importance).

|  | **10** | **30** | **50** | **100** | **150** | **200** | **250** | **300** | **350** | **400** | **450** | **500** | **550** | **600** |
| --- | --- | --- | --- | --- | --- | --- | --- | --- | --- | --- | --- | --- | --- | --- |
| **1** | SREBF1 | SREBF1 | SREBF1 | SREBF1 | SREBF1 | SREBF1 | SREBF1 | SREBF1 | SREBF1 | SREBF1 | SREBF1 | SREBF1 | SREBF1 | SREBF1 |
| **2** | SREBF2 | SREBF2 | SREBF2 | SREBF2 | SREBF2 | SREBF2 | TFAP2A | SREBF2 | TFAP2A | TFAP2A | TFAP2A | TFAP2A | TFAP2A | TFAP2A |
| **3** | HNF4A | TP63 | HNF4A | HNF4A | HNF4A | TFAP2A | SREBF2 | TFAP2A | SREBF2 | SREBF2 | SREBF2 | SREBF2 | SREBF2 | SREBF2 |
| **4** | TFAP2A | HNF4A | TP63 | TFAP2A | TFAP2A | HNF4A | HNF4A | HNF4A | HNF4A | HNF4A | HNF4A | HNF4A | HNF4A | HNF4A |
| **5** | TP63 | IRF2 | TFAP2A | TP63 | TP63 | TP63 | TP63 | TP63 | TP63 | TP63 | TP63 | TP63 | TP63 | TP63 |
| **6** | NR4A1 | FOXO4 | IRF2 | IRF2 | NR4A1 | ZNF217 | ZNF217 | ZNF217 | IRF2 | IRF2 | IRF2 | IRF2 | IRF2 | ZNF217 |
| **7** | IRF2 | NR4A1 | ZNF217 | FOXO4 | ZNF217 | IRF2 | IRF2 | IRF2 | ZNF217 | ZNF217 | ZNF217 | ZNF217 | ZNF217 | IRF2 |
| **8** | MYOD1 | TFAP2A | GATA1 | ZNF217 | IRF2 | NR4A1 | NR4A1 | NR4A1 | NR4A1 | NR4A1 | NR4A1 | NR4A1 | NR4A1 | NR4A1 |
| **9** | RARA | GATA1 | FOXO4 | NR4A1 | ARID2 | ARID2 | ARID2 | ARID2 | FOXO4 | FOXO4 | FOXO4 | FOXO4 | FOXO4 | FOXO4 |
| **10** | PPARA | PPARA | NR4A1 | ARID2 | FOXO4 | FOXO4 | FOXO4 | FOXO4 | ARID2 | ARID2 | ARID2 | ARID2 | ARID2 | ARID2 |

The figure below shows the average ROC AUC values of classification. The model was fitted with different numbers of estimators (shown in the x-axis).

**2. I recommend that the authors briefly describe each section in the introduction. This might help readers know the purpose of each section so that they can refer directly to that section for a detailed explanation.**

Thank the reviewer for the suggestion. We already describe the main findings of our manuscript in the last paragraph of the introduction, and now according to the reviewer’s suggestion, we also give section numbers here.

**3. On page 9, the second paragraph refers readers to figure 2E. I believe that the authors meant 2D.**

We thank the reviewer for the remark. We corrected it.

We thank the reviewer for useful comments and suggestions, and hope our answers addressed all their concerns.

### Reviewer #2

**Comments to the Authors:**

**The authors use public data on changes in the gene expression of cell lines treated with antiviral drug candidates and cell lines infected by SARS-COV-2, showing that similarities in the shifts of expression levels can be predictive of the effectiveness of drugs. For some drug candidate they then test a causal mechanism identified by the similarity-based analysis and find that the antivirals reduce cholesterol content of the plasma membrane and their effect can be reverted by cholesterol replenishment.**

**I find the second experimental part is a nice confirmation of the computational methods of the first part, and it is in this 2nd part that the main novelty of the paper lies. The computational methods used in the article were previously developed, so I don't see a methodological innovation there, except on the conceptual level that the authors used similarity and not dissimilarity of GE changes between drug-treated and infected cells to make drug effect predictions. In this sense i'm not sure PLoS Comp Biol is the best venue for this paper, but this is for the editors to decide. Since using public data anyhow in the 1st part of the article I think the authors could check their similarity based prediction for other viruses if data is available, ie. can drug effects be predicted for other viruses too based on similarity? Overall I found the article interesting and convincing for the specific hypothesis made re SARS-CoV-2, to make a general argument for similarity-based predictions though I find the sample size might be too small at this point.**

We thank the reviewer for the valuable comments and suggestions. We agree with the reviewer that our current manuscript supports our “signature similarity” principle only in the case of SARS-CoV-2 infection. While we discuss the possibility to extend this method to other virus infections, we think generalisation of this hypothesis is out of the scope of the current manuscript. However, according to the suggestions of the reviewer, we performed some preliminary analysis towards these directions, which we include in the following points.

**Some comments on figures and the text:**

**p6: AKT1 and MAPK1 are among the most connected nodes in PPI/gene regulation networks. Is this specific to sars-cov-2 at all? Comparison with other viruses could be useful here.**

AKT1 and MAPK1 are indeed the most connected nodes of the CARNIVAL inferred network of Figure 1C. As these two proteins are well characterised, hub nodes of signalling networks, this could be an artefact of the network contextualisation. However, there is literature evidence that these proteins play an important role in case of SARS-CoV-2 [[1]](https://paperpile.com/c/splD8M/KJdI) and MERS [[2]](https://paperpile.com/c/splD8M/C9jw) infections. According to the reviewer’s suggestions, we performed some additional literature review, and found evidence regarding the importance of these proteins / pathways also in other viral infections, like retroviruses [[3]](https://paperpile.com/c/splD8M/e4qE) and influenza A [[4]](https://paperpile.com/c/splD8M/u3Qq). We now cite these publications in the manuscript:

Key identified intermediate nodes AKT1 and MAPK1 were already connected to coronavirus infection (Kindrachuk et al. 2015; Bouhaddou et al. 2020) and other viral infections (Tian et al. 2015; Ehrhardt et al. 2007), also suggesting that the observed TF changes are initiated by the RIG-I like receptors, thus corresponding to the antiviral response of the host cell.

**On p11-12 the authors say the prediction performance based on gene expression (GE) similarities between infection and antivirals is 'reasonable'. It would be good to have a comparison with other studies where such predictions were validate, ideally not just in vitro. Are these ROC AUC values typical for such studies? Or higher?**

We agree with the reviewer, that comparing our method’s predictive performance can help to assess its usability. Our signature similarity-based method reached 0.75 ROC AUC for predicting effective antiviral drugs. In a recent study, Gysi et al. [[5]](https://paperpile.com/c/splD8M/jOCz) used a network-based approach to identify repurposable compounds against SARS-CoV-2. Their pipeline identified *in vitro* effective drugs with ~0.6 ROC AUCs, while drugs used in clinical trials (but not necessarily with clinical benefit) were predicted with ROC AUC ranging from 0.5 - 0.8 (using different network-based methods). In another recent study, [[6]](https://paperpile.com/c/splD8M/RwB7), the authors used chemical and sequence-based features (from drug and virus side) to predict repurposable antiviral drugs for new indications. Their different computational methods reached ~0.7 ROC AUC performance with leave-one-out cross-validation. These comparisons suggest that our prediction pipeline indeed reaches “reasonable performance”, and this is now also indicated in the main text.

In summary, our two different prediction approaches showed reasonable performance (comparable to studies based on network medicine and chemical similarity, (Morselli Gysi et al. 2021; Liang et al. 2020)), to predict drugs with in vitro anti-SARS-CoV-2 activity, and also highlighted the importance of previously discussed SREBF1/2 transcription factors.

**Fig3B: since there seems to be data on gene expression shifts for other viruses, could the similarity based prediction be applied to some of these? For instance can effectiveness of flu drugs be predicted from the similarity of GE shifts in drug-treated and infected cells? If so, this could be a further argument for the method.**

We agree with the reviewer, that generalisation of our “signature similarity” principle would be very interesting, and would definitely increase the impact of our study. Unfortunately, we are not aware of any other large scale drug screening dataset in case of other virus infections that currently could be used for a satisfactory benchmark of generalisation. However, we also found this question inquiring, thus we performed some preliminary analysis towards this direction.

Recently, a large scale CRISPR screen was performed [[7]](https://paperpile.com/c/splD8M/vhTG) to identify host dependency factors for influenza A infection. While our “signature similarity” principle was used for drugs in the current manuscript (effective drugs induce similar gene expression signature to viral infection), it can be extended to other perturbations, such as CRISPR KOs: if a perturbation-induced gene expression signature is similar to a virus infection-induced gene expression signature, than the virus replication can be inhibited using the given perturbation. To test this hypothesis, we tried to predict the targets identified by [[7]](https://paperpile.com/c/splD8M/vhTG) using the similarity between the CRISPR KO-induced gene expression signatures (from LINCS-L1000 screen) and influenza A-induced infection signature in Calu-3 cells (GSE37571). While the performance of our predictions was weaker than in case of SARS-CoV-2 drug signatures (probably highlighting differences between drug and CRISPR induced gene expression signatures), we were able to predict effective targets with ROC AUC 0.595. While we think these results are encouraging regarding the generalisation of the signature similarity principle, we also think that they are too preliminary (and also not directly connected to the current manuscript), so we do not intend to include them in the current manuscript. However, the reviewer can access it on the GitHub page of the manuscript (<https://github.com/comp-sys-pharm/SARS-CoV-2-cholesterol/blob/main/notebooks/6_Additional_analysis_CRISPR_KO_InfluenzaA_similarity.ipynb>).

**In Fig3/C what do the diamonds correspond to? (and also the intervals - CI50 and CI95? How were these intervals calculated?)**

We agree with the reviewer that the description of boxplots was not clear in our manuscript. Now we added the following text to the legend of Figure 3:

*“Boxplots represent the median (central line), first and third quartile (box), minimum and maximum non-outlier values (whiskers) and outliers (diamonds).”*

**In Fig4/C it's not clear to me what is meant by predictive probability exactly and why the highest values here are 0.1-0.2. This is a probability with respect to which model? How do these values compare to other drug effect prediction studies?**

We agree with the reviewer, that the values of predicted probabilities were not explained clearly enough in the manuscript. At first, the values on the x-axis of Figure 4C are predict**ed** probabilities of antiviral activity, instead of predict**ive** probabilities, which typo is now corrected in Figure 4C. These probabilities are the output of the Random Forest Classifier models, which is now indicated in the figure legend,

“*histogram of predicted probabilities of in vitro antiviral activity of LINCS-L1000 drugs (right panel, according to the Random Forest model)*”

and Methods:

Predicted probabilities of antiviral activity were also computed in each prediction and the mean of them was calculated for each drug (probabilities were summed for each drug and divided by the number of occurrences in validation sets).

The predicted probabilities are lower than 0.5, but this is only a consequence of the imbalanced nature of train / test datasets (47 effective vs. 4624 ineffective drugs in the whole dataset). Importantly, the used metrics (ROC AUCs) are using only the rankings of the predicted probabilities, thus are not affected by this imbalance. It is important to mention that the predicted probability by a random model would be 0.0101 (47 / (47+4624)), thus the reported predicted probabilities are higher by an order of magnitude.

**In Fig 4/D i can see well that there are a lot more intracellular cholesterol on the panels with the (predicted) effective drugs. However that there's less cholest. in the cell plasma membrane is less clear by the eye at least. We see too few cells in any case, so I was not convinced by this picture. I think the next panel makes the point much better.**

We agree with the reviewer that the time-course analysis (Figure 4E) shows the plasma membrane cholesterol depleting effect of amiodarone, chlorpromazine and MBCD better, especially with the statistical analysis. However we think that some representative images can help readers to better understand the whole phenomenon, so we also intend to keep these representative images in the manuscript.

**p14 and Fig4E: Just by eye the amiodarone curve looks very close and within the range of variation of the control. Somewhat surprised by the extremely low p-value. Is the p-value for the coefficient of the linear fit rather than on whether there's a difference wrt the control? Can the authors explain this? Maybe a significance test on the difference with DMSO would be better, as this one also shows a downward trend (by eye).**

We absolutely agree with the reviewer that the correct statistical test is a comparison with DMSO in this case. We indeed performed the following statistical test:

log2(PM/IC) ~ Time + Time : Drug + Exp linear model, where Time corresponds to elapsed time after drug treatment, Drug factor represents the used drug, using DMSO as reference level. Exp factor represents the (n=3) individual experiments.

We used the significance of interaction between Time and Drug factors (Time:Drug) to assess the (time-dependent) effect of drugs (compared to DMSO). Unfortunately, this was not correctly indicated in the figure legend, which is now corrected:

**: significant (p<0.001) interaction between drug treatment and elapsed time in linear model.*

Also, it is worth mentioning that we used an automated, high throughput confocal microscopy setup in these experiments, leading to a large number (>10,000) of observed cells, leading to the observed low p values.

**Fig5B: is the bar chart on the right an illustration or actual data? if the latter there should be error bars. If it's an illustration I am not sure it is needed.**

Figure 5B is only an illustration of the expected results of our cholesterol rescue experiments. We did not put data points and/or error bars into this schematic figure, not to confuse the readers. However we think this schematic figure can help to explain this complicated experimental setup, thus we intend to keep it in the manuscript.

**Minor comments:**

**0: remove paperpile links in file**

Thank the reviewer for the comment. We deleted the links.

**p4: COVID-19 disease burden. These are the *reported* numbers; real # of deaths likely to be higher. Refer to excess mortality estimates or say 'reported deaths'.**

Thank the reviewer for drawing our attention to the inaccuracy. We corrected it as follows (also adding the correct numbers for current date):

The newly emerged Severe Acute Respiratory Syndrome Coronavirus 2 (SARS-CoV-2), causing the coronavirus disease 2019 (COVID-19), has led to more than 420,000,000 reported infections and 5,500,000 reported deaths worldwide (Dong, Du, and Gardner 2020) until February 2022.

**p9: refers to Fig 2E, which doesn't exist. I assume it's Fig2D?**

We thank the reviewer for the remark. We corrected it.

**p15: I don't find Fig4A intuitive, specifically that the drugs (might) cause cholesterol depletion. I think this should be more explicitly shown somehow (eg. with arrows)**

According to the reviewer’s suggestion, now we extended the legend of Figure 4A the following way:

*(A) Schematic figure of the hypothesis that antiviral drugs block virus entry into cells by cholesterol depletion from plasma membrane, and are leading to a compensatory increased SREBF1/2 activity. Effects induced by viral infection are marked with black arrows (left side), while orange arrows represent drug-induced changes (right side).*

We thank the reviewer for useful comments and suggestions, and hope our answers addressed all their concerns.

### Reviewer #3

**Summary**

**The authors make a complete and through exploration and analysis to obtain the activity of pathways and TF in covid infected cells. Their pipeline investigates the pathways and TFs activities of different cells and compare with the activity of drug induced cells, is very clear and straightforward.**

**They revealed that the similarity of the TF activities in infected cells and drug induced cells are an important feature to determine the efficiency of the drug treatments for SARS-coV-2 in vitro (although not true for all effective drugs). They as well demonstrated quantitatively that the similarity to infection signatures is good to predict effective drugs. This result is important since it can be explored in the search for treatments of another virus.**

**The main result from the comparison of the T.F. activities, infected vs drug treated cells, is that regardless of their similarity, for some clusters of drugs, they observe a high activation of key regulators of cholesterol, SREBFs. Which is surprising given that high cholesterol synthesis can stimulate viral entry. They hypothesize a mechanistic scenario for this contra intuitive result: The anti-SARS-CoV-2 drugs decrease plasma membrane cholesterol, which in turn activate SREBFs TFs.**

**To prove this, they performed a series of experiments to demonstrate the role of the membrane cholesterol in the antiviral effect of 3 different drugs (efficient drugs against SARS-coV-2).**

We thank the reviewer for the positive comments regarding our work.

**Comments and questions:**

**-I did not have clear what was the input of the Random Forest Classification, since one result obtained from it is the importance of HNf4A, TFAP2A and TP63 transcription factors, but they are not presented in Fig.1B nor Fig.2B as to have available their activities under drug induction. The authors could explain why the lack of such data.**

For the Random Forest Classification model, we used drug-induced transcription factor activities, calculated by the DoRothEA tool [[8]](https://paperpile.com/c/splD8M/5I2q) as input features. DoRothEA calculated activities for 288 transcription factors, including HNF4A, TFAP2A and TP63. However, for easier interpretation of TF activity heatmaps (Figure 1B and 2B), we showed only the most strongly affected transcription factors in the heatmaps. Importantly, clustering of drugs (Figure 2B) was performed using all transcription factor activities, and in Figure 2C all transcription factors are shown in the scatterplot. We already mentioned this filtering for transcription factors in the legend of Figure 1, but now we added the following sentences to the legend of Figure 2 and Methods:

*Only selected transcription factors (corresponding to Figure 1B) are shown.*

In transcription factor activity heatmaps (Figure 1B and Figure 2B), only selected transcription factors (absolute normalised enrichment score > 4 in SARS-CoV–2 infection signatures) are shown.

While HNF4A, TFAP2A and TP63 transcription factors are not shown in the heatmaps (as they are not among the most differentially activated transcription factors), their activities are strongly increased, as shown in the histogram below - thus corresponding to their importance in the Random Forest model.

**--They were able to demonstrate the role of membrane cholesterol importance for the drug antiviral effect in only 1 out of 3 investigated drugs. Even so, they show (Fig.4C) that the depletion of membrane cholesterol is sensitive to the elapsed time after drug induction, therefore time can be a key parameter, specially in pre-treatment of cells, for the cholesterol localization and its effect once the cells are infected. Therefore, this encourages to a further study of the mechanism of the antiviral effect of drugs over time and the relationship with cholesterol.**

We agree with the reviewer that time is an important factor in cholesterol depletion and/or resynthesis, thus also in the antiviral effect of cholesterol depleting drugs. As we discuss in the following point, while chlorpromazine exerts antiviral effect in case of co-treatment, we were not able to measure antiviral effect in case of pre-treatment. This suggests that optimal timing of drug treatment, viral infection and cholesterol rescue can strongly influence the antiviral effect. We agree with the reviewer that further studies are needed to understand these relationships, but we think they are out of the current scope of the manuscript.

**--Do the authors have an explanation on why Chlorpromazine shows similar viral copies as in the control (Fig. 5C), with and without cholesterol replenishment?**

As the reviewer noted (previous point), time is definitely an important factor regarding the antiviral effect, and also cholesterol depletion of drugs. Chlorpromazine shows a strong antiviral effect in case of co-treatment (Figure 5A, bottom left panel), while in case of pre-treatment (and cholesterol rescue), we were not able to observe this antiviral effect (Figure 5C). Regulation of the plasma membrane cholesterol content is a complicated, dynamic process, thus it is possible that the strong cholesterol depletion effect of chlorpromazine can lead to feedback mechanisms in case of the pre-treatment. We agree with the reviewer, that analysing the time scale of the drug effect (especially in case of chlorpromazine) is a very interesting question, however we think it needs further studies.

**Minor note:**

**-Numbering in Fig.4, the numbering A and B are cut and the x-label od 4.C is missing (is the elapsed time in minutes)?**

Thank the reviewer for identifying these problems. We corrected the numberings and the missing labels.

**I appreciate the complete work done in the paper: it includes a curation of previous experimental data, network analysis, statistical analysis, and finally, they performed well focused experiments to try to demonstrate their hypothesis.**

**Overall, I think the article presents a good study and demonstrates the important role of plasma membrane cholesterol in the antiviral effect of some drugs against SARS-Cov-2. It also gives good information (and technique) to evaluate the role of other factors and mechanism for drug and for infection, as they present the activities of many other pathways and transcription factors. This can be helpful not only in the focus of the role of cholesterol but be complementary of other studies, as the mechanisms of other repurposing drugs.**

**Hence, with the above major comment and a few minor comments addressed I found that this manuscript deserves be published in PLOS Computational Biology.**

We thank the reviewer for the positive feedback and valuable comments, especially highlighting the importance of our methods in further research of other drug repurposing studies. We hope our response answered their questions.

### Reviewer #4

**Barsi et al performed computational analyses on transcriptomics data from in vitro SARS-CoV-2 infected cell lines and from cell lines treated with drugs showing anti-SARS-CoV-2 activity. Using functional genomics study and machine learning, the authors identified SREBF1 and SREBF2 transcription factors that regulate lipid metabolism are activated by effective drugs. The authors hypothesized and further showed drug-induced decreased level of plasma membrane cholesterol via depleted cholesterol metabolism has anti-viral effect. Their experimental validations showed amiodarone, a drug that decreases plasma-membrane cholesterol content is a potential repurposing drug candidate to combat SARS-CoV-2.**

**This is an interesting study and the work connects depleted plasma membrane cholesterol induced by drugs exert anti-SARS-CoV-2 activities and open a new door to repurpose existing drugs to combat COVID-19.**

We thank the reviewer for the positive feedback and for the minor suggestions which aim to improve the quality of our manuscript.

**I have no major concern on the results on this work. The work is deemed publishable with few minor concerns being addressed:**

**(1) There is a number of reported works on the role of cholesterol (including SREBF) with COVID-19, the authors should mention and discuss some of them to provide a clearer picture how the present work fills the gap in our understanding and treatment of COVID-19.**

As these points both affect the discussion, we answer them together below.

**(2) Although the authors showed depleted plasma membrane cholesterol is essential for anti-viral activities and described increased SREBF1/2 as a compensatory consequence, the authors might need to provide further elaboration on the following points:**

**(i) Is inhibition of SREBF1/2 an adaptive response of infected cells to restrict the supply of cholesterol to prevent viral entry (i.e., a protective mechanism of infected cells against virus)?**

We thank the reviewer for highlighting some important aspects to discuss. According to the suggestions, we extended our discussion the following way:

Cholesterol depletion of plasma membrane can reduce SARS-CoV-2 infection (S. Wang et al. 2020; Sanders et al. 2021), and decreasing SREBFs activity (and cholesterol synthesis) can be also part of the physiological, interferon-induced antiviral response of the host cell (O'Neill 2015; York et al. 2015; Blanc et al. 2011). Recently, inhibitors of the SREBFs - DNA interaction were found to exert antiviral effects (Yuan et al. 2019) and CRISPR based knock-out of the SREBF pathway members also led to SARS-CoV-2 resistant phenotype (Wang et al. 2021), suggesting that inhibition of SREBFs could be beneficial in case of SARS-CoV-2 infection. In contrast, we found increased activity of SREBFs in case of several effective drug induced gene expression signatures.

**(ii) Is increased SREBF1/2 by effective drugs a homeostatic (or protective) response of cell to counter cytotoxic effect of effective drugs that lead to decrease plasma membrane cholesterol?**

Cholesterol depletion of plasma membrane can definitely induce cell death [[9]](https://paperpile.com/c/splD8M/8GJd), thus increased resynthesis can be a homeostatic mechanism to counteract it. Further studies would be needed whether the cytotoxicity of effective drugs is influenced by SREBF activity.

**(3) The usage of the term “drug signature” is confusing as it can be understood as drug-induced gene expression patterns or chemical signature of drug. The authors need to define this term more explicitly in the introduction.**

We agree with the reviewer and we changed “drug signature” to “drug-induced signature” which we think makes it clear that we refer to the drug-induced gene expression pattern instead of the chemical signature of the drug.

**(4) Revise subtitle of section 2.5 to “Supplementing cholesterol reverse anti-SARS-CoV-2 activity of amiodarone”.**

Thank the reviewer for the suggestion that makes this section title more understandable. We changed the section title according to the recommendation.

### References

1.	[Bouhaddou M, Memon D, Meyer B, White KM, Rezelj VV, Correa Marrero M, et al. The Global Phosphorylation Landscape of SARS-CoV-2 Infection. Cell. 2020. doi:](http://paperpile.com/b/splD8M/KJdI)[10.1016/j.cell.2020.06.034](http://dx.doi.org/10.1016/j.cell.2020.06.034)

2.	[Kindrachuk J, Ork B, Hart BJ, Mazur S, Holbrook MR, Frieman MB, et al. Antiviral potential of ERK/MAPK and PI3K/AKT/mTOR signaling modulation for Middle East respiratory syndrome coronavirus infection as identified by temporal kinome analysis. Antimicrob Agents Chemother. 2015;59: 1088–1099.](http://paperpile.com/b/splD8M/C9jw)

3.	[Tian J, Zhang X, Wu H, Liu C, Li Z, Hu X, et al. Blocking the PI3K/AKT pathway enhances mammalian reovirus replication by repressing IFN-stimulated genes. Front Microbiol. 2015;6: 886.](http://paperpile.com/b/splD8M/e4qE)

4.	[Ehrhardt C, Wolff T, Pleschka S, Planz O, Beermann W, Bode JG, et al. Influenza A virus NS1 protein activates the PI3K/Akt pathway to mediate antiapoptotic signaling responses. J Virol. 2007;81: 3058–3067.](http://paperpile.com/b/splD8M/u3Qq)

5.	[Morselli Gysi D, do Valle Í, Zitnik M, Ameli A, Gan X, Varol O, et al. Network medicine framework for identifying drug-repurposing opportunities for COVID-19. Proc Natl Acad Sci U S A. 2021;118. doi:](http://paperpile.com/b/splD8M/jOCz)[10.1073/pnas.2025581118](http://dx.doi.org/10.1073/pnas.2025581118)

6.	[Liang H, Zhang L, Wang L, Gao M, Meng X, Li M, et al. Repositioning Drugs on Human Influenza A Viruses Based on a Novel Nuclear Norm Minimization Method. Front Physiol. 2020;11: 597494.](http://paperpile.com/b/splD8M/RwB7)

7.	[Li B, Clohisey SM, Chia BS, Wang B, Cui A, Eisenhaure T, et al. Genome-wide CRISPR screen identifies host dependency factors for influenza A virus infection. Nat Commun. 2020;11: 164.](http://paperpile.com/b/splD8M/vhTG)

8.	[Garcia-Alonso L, Holland CH, Ibrahim MM, Turei D, Saez-Rodriguez J. Benchmark and integration of resources for the estimation of human transcription factor activities. Genome Res. 2019;29: 1363–1375.](http://paperpile.com/b/splD8M/5I2q)

9.	[Gu L, Saha ST, Thomas J, Kaur M. Targeting cellular cholesterol for anticancer therapy. FEBS J. 2019;286: 4192–4208.](http://paperpile.com/b/splD8M/8GJd)
